# Google Searches and Suicide Rates in Spain, 2004-2013: Correlation Study

**DOI:** 10.2196/10919

**Published:** 2020-04-13

**Authors:** Alberto Jimenez, Miguel-Angel Santed-Germán, Victoria Ramos

**Affiliations:** 1 Instituto de Salud Carlos III, ISCIII Information and Communication Technologies Unit Madrid Spain; 2 The National Distance Education University (UNED) Madrid Spain; 3 Instituto de Salud Carlos III, ISCIII Telemedicine and Health Research Unit Madrid Spain

**Keywords:** suicide, big data, infodemiology, infoveillance, incidence, help-seeking behaviors, searching behavior, early diagnosis

## Abstract

**Background:**

Different studies have suggested that web search data are useful in forecasting several phenomena from the field of economics to epidemiology or health issues.

**Objective:**

This study aimed to (1) evaluate the correlation between suicide rates released by the Spanish National Statistics Institute (INE) and internet search trends in Spain reported by Google Trends (GT) for 57 suicide-related terms representing major known risks of suicide and an analysis of these results using a linear regression model and (2) study the differential association between male and female suicide rates published by the INE and internet searches of these 57 terms.

**Methods:**

The study period was from 2004 to 2013. In this study, suicide data were collected from (1) Spain’s INE and (2) local internet search data from GT, both from January 2004 to December 2013. We investigated and validated 57 suicide-related terms already tested in scientific studies before 2015 that would be the best predictors of new suicide cases. We then evaluated the *nowcasting* effects of a GT search through a cross-correlation analysis and by linear regression of the suicide incidence data with the GT data.

**Results:**

Suicide rates in Spain in the study period were positively associated (*r*<-0.2) for the general population with the search volume for 7 terms and negatively for 1 from the 57 terms used in previous studies. Suicide rates for men were found to be significantly different than those of women. The search term, “allergy,” demonstrated a lead effect for new suicide cases (*r*=0.513; *P*=.001). The next significant correlating terms for those 57 studied were “antidepressant,” “alcohol abstinence,” “relationship breakup” (*r*=0.295, *P*=.001; *r*=0.295, *P*=.001; and *r*=0.268, *P*=.002, respectively). Significantly different results were obtained for men and women. Search terms that correlate with suicide rates of women are consistent with previous studies, showing that the incidence of depression is higher in women than in men, and showing different gender searching patterns.

**Conclusions:**

A better understanding of internet search behavior of both men and women in relation to suicide and related topics may help design effective suicide prevention programs based on information provided by search robots and other big data sources.

## Introduction

### Background

According to the World Health Organization (WHO) projections, by 2030, there will be 1,007,000 deaths by suicide, making suicide the 15th leading cause of death globally and accounting for 1.4% of all deaths [1]. Despite the common idea that suicide is more prevalent in high-income countries, about 75% of suicides worldwide occur in low- and middle-income countries. In general, suicide rates are lower among people aged <15 years and >70 years [2].

With a rate of 10 cases every day, suicide is the leading cause of unnatural death in Spain, producing more than twice as many deaths than traffic accidents, 7 times more deaths than workplace accidents, and 70 times more deaths than domestic violence. It is also the leading cause of death among men aged 20 to 24 years [3].

The incidence of suicide in a society depends on a range of factors, of which clinical depression is a particularly common cause [4]. Substance abuse, severe physical disease, and disability are also recognized causes of suicide. Countries in Eastern Europe and East Asia have the highest suicide rate in the world. The region with the lowest suicide rate is Latin America. Gender differences also play a significant role: Among all age groups in most parts of the world, females tend to show higher rates of reported nonfatal suicidal behavior and males have a much higher rate of completed suicides.

#### Availability of Google and the Internet

As Howe [5] reports, the internet was the result of some visionary thinking by people in the early 1960s who saw great potential in allowing computers to share information on research and development in scientific and military fields. There is the common idea that widespread media coverage of specific methods of suicide may induce copycat deaths and initiate changes in the popularity of certain methods since at-risk individuals may use the internet to research particular methods of suicide that can be more lethal than the commonly used methods [6]. It is unclear whether the information obtained on the internet is reducing the risk of suicide or contributing to suicide promotion; there is evidence to suggest that the internet may facilitate suicide in various ways [7], but the influence of the internet on the incidence of suicide is not well known. On the contrary, efforts to carry out epidemiological monitoring of suicide are hampered by gaps in data availability. At present, the lag time for reporting data is 3 years for the Centers of Disease Control and Prevention (CDC) in the United States, ≥5 years for the WHO [8], and about 3 years for the Instituto Nacional de Estadística (INE, National Statistics Institute) in Spain.

#### Using Google Search Totals to Predict Social Trends

Increasingly, the volume of internet searches is being used as a social indicator (eg, in the field of epidemiology), and recently, this method has been applied to studies on suicide. We can establish a chronology of studies that began to use internet search volumes following the study of Choi and Varian [9], who reviewed the pioneering studies that suggested that web search data are useful for forecasting in various fields. In economics, the first such study was performed by Ettredge et al [10], who examined the association between search volumes and unemployment rates in the United States. In the same year, Cooper et al [11] described the use of internet search volumes for cancer-related topics. Since then, there have been several papers that have examined web search data in numerous ﬁelds.

In the ﬁeld of epidemiology, Eysenbach [12]—as the initiator—and Ginsberg et al [13] showed that search data could help predict the prevalence of inﬂuenza-like diseases by finding a positive relationship between the number of influenza-related search queries and pneumonia and influenza mortality. These papers were widely publicized and stimulated several further ﬁndings in epidemiology, including those by Brownstein et al [14], Hulth et al [15], Pelat et al [16], and Valdivia and Monge-Corella [17].

In the ﬁeld of economics, Choi and Varian [9] showed how Google Search Insights data could be used to predict some economic metrics including initial claims for unemployment, vacation destinations, and automobile demand. Askitas and Zimmermann [18] and Suhoy [19] inspected unemployment data in the United States, Germany, and Israel. Guzman [20] examined Google data as a forecaster of inﬂation, pointing out that the Google Inflation Search Index (GISI) indicator is a good way of measuring inflation. Baker and Fradkin [21] have used Google search data to examine how job search activity was influenced by policies on unemployment payment extensions. Radinsky et al [22] and Preis et al [23] examined the use of search data for measuring consumer confidence, and Vosen and Schmidt [24] studied consumption and retail sales metrics.

Shimshoni et al [25] verified the predictability of Google Trends data, showing that substantial quantities of search terms are greatly predictable using simple seasonal statistical methods. Goel et al [26] offered a useful survey of work in this area, revealing some of the limitations of web search data. As they pointed out, obtaining search data is easy and often helpful in making predictions but it may not provide significant increases in predictability.

Recent studies have shown the usefulness of new methodologies known as *Infoveillance*, *Infodemiology*, or *Digital Disease Surveillance*. For example, Adler et al [27], through projections of known correlations, identified various states in India with poor surveillance of the incidence of suicide or states with limited or no access to the internet.

#### Forecasting Suicide

Work on suicide has predominantly focused on traditional forms of media, particularly surrounding the issue of suicide contagion.

Daine et al [28] conducted a systematic review investigating the influence of the internet on self-harm and suicide in young people. They provided evidence of both positive influences, such as web-based media being used as a form of support, and negative influences, such as internet addiction, cyberbullying, and the internet being a source of information on suicide and self-harm. Mok et al [7] expanded on previous work by focusing explicitly on suicide-related internet use. They define suicide-related internet use as the “use of the Internet for reasons relating to an individual’s own feelings of suicide” [7]. This paper summarized and assessed the existing work on not only the influence of suicide-related internet use but also its nature by presenting the main findings and discussing the types of studies that have been conducted, their strengths and limitations, and recommendations for future research. These findings are reported in Textbox 1.

In this study, we have focused on the topic, “Suicide-related internet search trends can provide an indicator of suicide risk in a population” in Textbox 1. According to Mok et al [7], most of the 9 articles give credence to a link between suicide-related search activity and suicide rates.

Some papers studied the correlation between search terms such as “suicide” and “depression” [8] in searches and news reports [29], or between searches and unemployment rates [30]; therefore, we have excluded this kind of semantic or mass media correlation, focusing only on the correlation between search terms and actual death rates reported by official institutions (ie, the INE for the 2004-2013 period). We did not find Chen’s 2013 paper reported by Mok et al [7] and Gunn and Lester [31]; as such, we interpreted this as a citation error in the Mok et al [7] paper. Therefore, we finally used 6 articles (Table 1) that studied a total of 57 terms, of which 14 do not return results in Spanish in Google Trends for the period studied in Spain (Table 2).

**Main findings of the literature on suicide-related internet use [7].**

- Use of the internet to search for suicide-related content:
  - Suicide-related internet search trends can provide an indicator of suicide risk in a population (number of articles, n=9).
  - Users conducting suicide-related searches typically access scientific information and community resource websites (n=1).
- Use of the internet to express suicide-related feelings (n=7)
- Suicide-related internet use and suicidal behavior:
  - The internet may facilitate suicide in various ways (n=17).
  - Internet-related suicides are rare when compared with overall suicides (n=1).
  - There is no evidence of increased suicidal behavior in response to a suicide on a web-based forum (n=1).
- Suicide-related internet use and suicidal ideation:
  - Individuals who engage in suicide-related internet use report higher levels of suicidal ideation (n=4).
  - There are mixed findings regarding the influence of suicide-related internet use on suicidal ideation over time (n=6).
  - Informal web-based suicide communities can maintain suicidal feelings (n=1).
- Role of the internet in suicide prevention:
  - Informal web-based suicide communities can function as support groups (n=1).
  - Web-based suicide forums staffed by trained volunteers can have positive effects (n=3).
  - Professional web-based interventions can reduce suicidal ideation (n=2).

**Table 1:** Previous studies on the topic, “Suicide-related internet search trends can provide an indicator of suicide risk in a population,” according to Mok et al [7]; the terms from this topic were tested in Spanish.

Year	Authors	Title	Region of study	Period	Language	Data	Studio unit	Statistical method
2010	McCarthy [8]	Internet monitoring of suicide risk in the population	United States	2004-2007	English	Centers for Disease Control and Prevention (CDC), United States	Year	One-way analysis of variance with Tukey-Kramer post-hoc analysis
2011	Sueki [32]	Does the volume of Internet searches using suicide-related search terms inﬂuence the suicide death rate: Data from 2004 to 2009 in Japan	Japan	2004-2009	Japanese	Demographic statistics released by the Ministry of Health, Labour, and Welfare	Month	Cross-correlation
2011	Yang et al [33]	Association of internet search trends with suicide death in Taipei City, Taiwan, 2004–2009	Taipei City, Taiwan	2004-2009	Chinese (traditional)	Department of Health, Taiwan	Month	Cross-correlation, multiple linear regression with a stepwise method
2012	Hagihara et al [34]	Internet suicide searches and the incidence of suicide in young people in Japan	Japan	2004-2010	Japanese	Statistics and Information Department of the Japanese Ministry of Health, Labour and Welfare	Month	Cross-correlation
2013	Gunn and Lester [31]	Using google searches on the internet to monitor suicidal behavior	United States	2009	English	McIntosh and Drapeau [35]	Month	Pearson correlations
2014	Bruckner et al [36]	A time-series analysis of google searches for suicide and the risk of completed suicide in England and Wales, 2004–2010	England and Wales	2004-2010	English	Publicly available database	Month	Time-series routines

**Table 2:** Terms used in previous studies with their Spanish translation.

Paper (English) search term		Spanish translation
**Bruckner et al [36]**		
	Depression and help^a^	depresión y ayuda
	Suicide and depression	suicidio -gran + depresion -gran
	Suicide and help^a^	suicidio y ayuda
	Suicide and methods	suicidio metodos
**Gunn and Lester [31]**		
	Commit suicide	suicidarse
	How to suicide^a^	cómo suicidarse
	Suicide prevention^a^	prevención del suicidio
**Evans [37]**		
	A suicide	suicida -ataque -escuadron -fuga -comico -reportero -tango -letra -extremoduro
	Bulletin board system on suicide^a^	BBS^b^ sobre el suicidio
	Depression suicide^a^	depresión suicida
	Hydrogen sulﬁde	sulfuro de hidrógeno + sulfuro de hidrogeno
	Hydrogen sulﬁde suicide^a^	sulfuro de hidrógeno suicidio
	Sites on suicide^a^	sitios sobre suicidio
	Suicide by jumping^a^	suicidio saltando
	Suicide hydrogen sulﬁde^a^	suicidio por sulfuro de hidrógeno + suicidio por sulfuro de hidrogeno
	Suicide methods	maneras de suicidarse
	Suicide rates^a^	tasas de suicidio
**McCarthy [8]**		
	Teen suicide	suicidio adolescente
**Hagihara et al [34]**		
	Abuse	abuso
	Alcohol	alcoholismo
	Alcohol abstinence	dejar alcohol
	Allergy	alergia
	Antidepressant	antidepresivo
	Anxiety disorder	trastorno de ansiedad
	Asthma	asma
	Bipolar disorder	bipolar -pol -letra -chiguire -cancion
	Cáncer	cancer -horoscopo
	Charcoal burning	carbón vegetal + carbon vegetal
	Chronic illness	enfermedad cronica
	Complete guide of suicide^a^	guía completa de suicidio
	Divorce	divorcio
	Domestic violence	violencia domestica + violencia doméstica
	Drunkenness	emborracharse
	Hanging	colgarse
	Headache	dolor de cabeza
	Hypnotics	somniferos
	Illicit drugs	drogas
	Insomnia	insomnio -filmaffinity -la -pelicula -wow -dun
	Job	trabajo
	Jumping from a height^a^	saltar desde altura
	Lawsuit	demanda judicial
	Major depression	depresión mayor + depresion mayor
	Marriage	matrimonio
	Pain	dolor
	Psychiatric service^a^	servicio psiquiátrico
	Relationship breakup	ruptura amorosa
	Religious belief	creencias religiosas
	Schizophrenia	esquizofrenia
	Social beneﬁts	ayuda social
	Social welfare	bienestar social
	Stock market	bolsa de valores
	Stress	estres -bancos + estrés -bancos
	Taiwan economy	economia + economía
	Unemployed + lost job	paro; desempleo
**Bruckner et al, Evans, Hagihara et al [34,36,37]^c^**		
	Suicide methods	maneras de suicidarse
**Bruckner et al, Yang et al [33,36]^c^**		
	Depression	depresion -meseta -gran + depresión -meseta -gran
**McCarthy, Bruckner et al, Hagihara et al, Yang et al [8,33,36]^c^**		
	Suicide	suicidio

^a^Terms for which Google Trends returned the result, “your search does not return enough data to show results.”

^b^BBS: bulletin board system.

^c^Several studies evaluating the same term.

### Objectives

The study has two objectives: (1) It evaluates the correlation between suicidal rates released by the INE and internet search trends in Spain reported by Google Trends for 57 suicide-related terms representing major known risks of suicide; these terms have already been tested in previous scientific studies systematized by Mok et al [7] (topic “Suicide-related internet search trends can provide an indicator of suicide risk in a population”). (2) It examines the differential association between male and female suicide rates published by the INE and internet searches related to the aforementioned 57 terms. The study included data from 2004 to 2013, as this was the maximum period for which relevant data were available from the INE and Google Trends.

## Methods

In this section, we have addressed two issues: (1) how Google presents the results of search volume and how those results are normalized over time and in different geographical areas and (2) presentation of the variables we worked with—the expressions or terms used whose search volumes are reported by Google Trends and suicide rates (globally and segregated by gender) provided by the INE.

### Google Trends

Google Trends provides a time-series index of the volume of queries users entered into Google in a given geographic area. Wikipedia explains it as follows [38]:

Google Trends is a public web facility of Google Inc., based on Google Search, that shows how often a particular search-term is entered relative to the total search-volume across various regions of the world, and in various languages.

Although Google Trends does not show the absolute number of searches, it calculates a query share for a search term. This means that Google calculates the number of searches for a given term as a proportion of the total number of searches in each location at a given time. These calculations are then normalized to a Google Trends *Relative Search Volume* (RSV) index between 0 and 100, where an RSV index of 100 designates the date when there was the highest amount of search activity for that given term. Thus, a search index of 40 equates to 40% of the most intense search activity in the selected country at a given period.

Thus, the RSV index is a way to normalize (from 0 to 100) the query share that is the total volume of queries of the search term in question within a particular geographic region divided by the total number of searches in that region for the period under review. The maximum percentage of consultation in the specified time period is normalized to 100, and the other measures for that period of time are calculated relative to this value.

Google Trends also allows for the comparison of the relative volumes of blocks of searches for up to 5 terms or phrases. In this case, the RSV of other terms that did not reach the peak of 100 is normalized to the 100 value of the term with the highest search volume of the 5 terms of phrases in the block. However, in our work, terms were consulted one by one.

**Figure 1:**
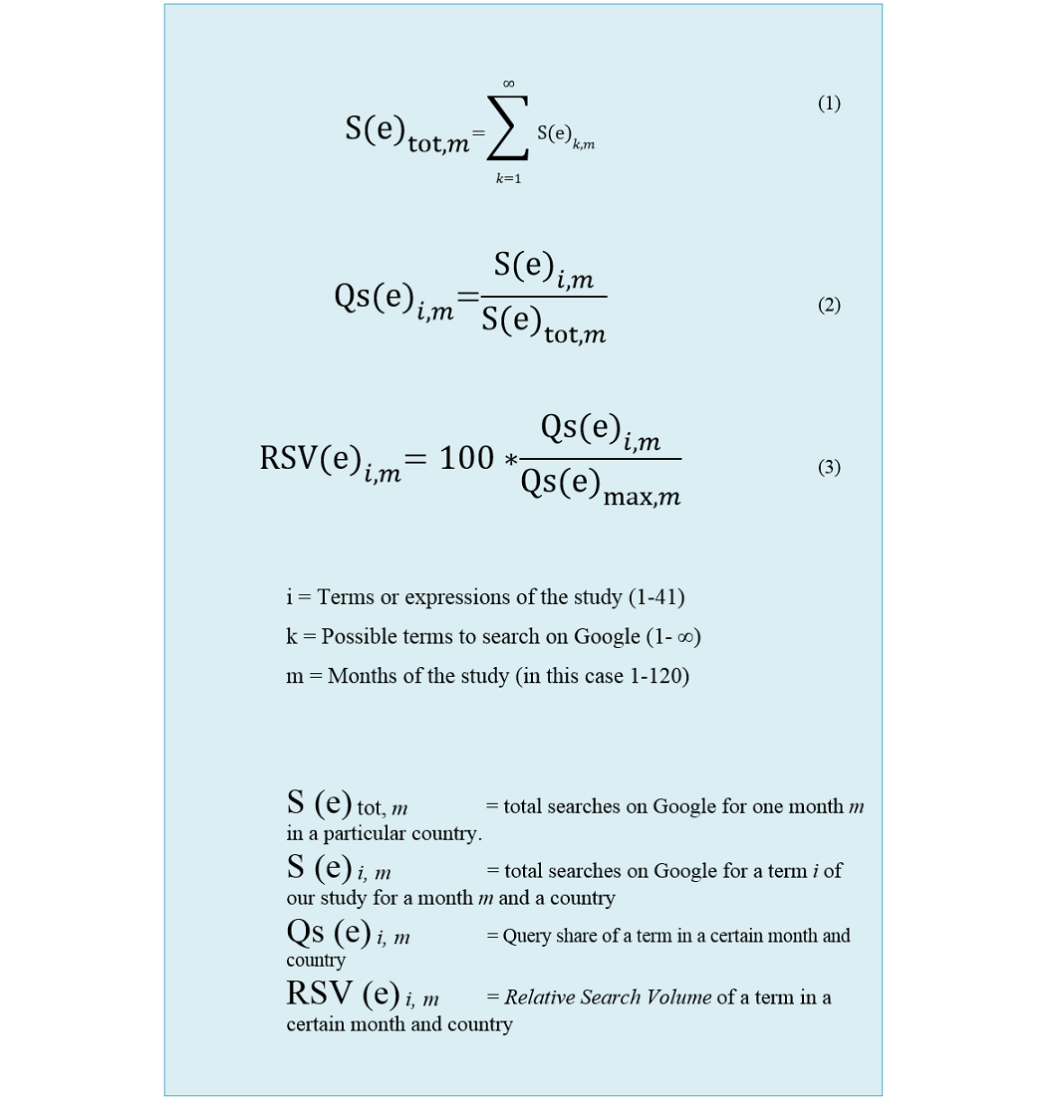
Mathematical formulation of how Google Trends operationalizes its monthly relative search volume for a particular term. RSV: relative search volume.

It is interesting to point out that although, according to Google Scholar, more than 10,000 scientific papers used or mentioned Google Trends service, we did not find any mathematical formulation of how the RSV value was calculated or operationalized by Google Trends. Therefore, we proposed a tentative mathematical formulation of how this value is calculated (Figure 1).

In short, Google Trends calculates the number of searches as percentages (formula 2 of Figure 1) based on the total searches in a month (formula 1 of Figure 1), normalizes the series allocated to the highest value (ie, the value of 100), and scales all other values accordingly (formula 3 of Figure 1).

### Variables

#### Search Term Variables Group

As variables, 57 query terms (Table 2) have been used that relate to suicidal ideation studied in the 6 articles mentioned in Table 1. These terms were translated into Spanish with the help of the website WordReference [39]; note that for cases in which the original language is different from the language of the articles (ie, English), this meant a third translation, as some of the papers were in Japanese and traditional Mandarin Chinese, which can be a significant semantic shift.

Queries to Google Trends are not case sensitive but are diacritical mark sensitive, so Google Trends has different results (eg, for “enfermedad cronica” (chronic illness) than for “enfermedad cronica + enfermedad crónica” [written with Spanish accent]).

Google queries are “broad matched” in the sense that queries such as “great depression” are counted in the calculation of the query index for “depression,” which is why we mentioned above that when searching for a term, we should look up what related queries pop out to exclude unwanted terms by placing a dash before them, as required by the Google Trends interrogation syntax. In addition, we have performed a back translation procedure to confirm the accuracy of the translation.

In the related searches, we found terms that did not include the one we were searching for; this is because Google showed other terms that were searched for in the same searching session as the one we were interested in, so we included that term preceded by a hyphen after ours to exclude this spurious concept out of our dataset. In this regard, we devised a set of terms (Table 2) to search in Google Trends; 14 returned no results for their Spanish translation (marked with “no” in the results column in Table 2), and the term “suicide methods” occurred 3 times in previous studies, so duplicates were removed. Therefore, in our final analysis, only 41 terms were included.

We performed an individual search for each of these terms in Spanish, and Google Trends returned 120 values, one for each month of the study period. In each series, there was one term with a value of 100 and the remaining were presented as percentages in reference to this.

Our search was limited to Spain for the period from January 1, 2004, to December 31, 2013, and the final configuration of the search for each term was as follows:

https://www.google.es/trends/explore?date= 2004-01-01%202013-12-31&geo=ES&q=”term”.

It is worth mentioning that Google Trends data were computed using a sampling method, and therefore, the results vary within minutes.

#### Group Suicide Rate Variables Collected by the Spanish National Statistics Institute

The variables that we used for correlations are the absolute actual suicide rates of Spain (around 4000 deaths per year) reported by the INE, the official organization in Spain that collects statistics on demography, economy, and Spanish society. We have obtained this information through the National Epidemiology Center, which is part of the Instituto de Salud Carlos III, a public research center of the Government of Spain. This information was segregated into totaled data for men and women; Google Trends data were not segregated in this manner.

The period that collected data for was from January 2004 to December 2013, which is, as mentioned earlier, the maximum period covered by both data sources, Google Trends and the INE, at the time of study.

### Statistical Analysis

Data were analyzed using the statistical package IBM SPSS Statistics, version 22 (IBM Corporation). The Pearson correlation coefficient was used to assess a possible monthly correlation between suicidal rates and Google Trends RSV data for the search terms that we defined. Next, we performed a multiple linear regression analysis to propose an explanatory-predictive model of the variance of the suicide rates variable.

## Results

### Results of Objective 1: Correlation Between Suicidal Rates and Internet Searches

With regard to the first objective of our study, the values for correlation between suicide-related terms and suicide rates for Spain from Google Trends data are shown in Table 3 after calculating the Pearson correlation coefficient. We centered moderate or superior results of correlation values according to Evans’ study [37], as detailed in Table 4, with a significance value *P*<.05 (in italics). Since the study terms are Spanish translations of ones already studied in English, Japanese, and Mandarin in the mentioned studies, we devised a *Reference* column indicating the previous study and a *Correlation* column to indicate the presence of a correlation according to the original study, with values *yes*, *insufficient*, or *no*.

A linear regression analysis (steps forward) was performed; predictors included all variables (search terms) that demonstrated a significant correlation with previous suicide rates collected by the INE and had an *r*>0.2. These are the terms in Table 3 that have at least one *P* value with a significant correlation in the men, women, or total columns. Table 4 presents the explanatory-predictive model.

Overall, the model predicts a significant percentage of variance (adjusted *r*^2^=0.387) of the suicide variable. The term “unemployment” translated as “paro” in Spanish has a high beta value and a positive sign, whereas the term “unemployment” translated as “desempleo” has a lower and negative value. This may seem contradictory because both terms are, a priori, synonyms. However, searches of the term “desempleo” could be carried out, in greater proportion, by people seeking information related to the official term “unemployment benefits and aid” offered by the Spanish Government, while the term “paro,” which is used more colloquially, may be associated with searches carried out by people who are suffering due to “unemployment.” This may explain why searches for that term are positively associated with the incidence of suicide committed in Spain between 2004 and 2013.

Regarding the beta value for “headache,” which has a high and negative correlation with the variable “incidence of suicide,” it could be argued that people who search for headache (a condition that can be associated with a wide variety of medical conditions) do so with an intent of self-care, which is contrary to the intention of committing suicide.

**Table 3:** Correlation coefficient obtained for each Spanish term related to suicide, and suicide rates for men and women in Spain in the 2004-2013 period. Significant values (ie, *P*<.05) are italicized.

Translated search term (original in Spanish)	Monthly correlation: GT^a^ RSV^b^/INE^c^ suicide rates (2004-2013)						Previous studies	
	Total		Men		Women		Reference	Correlation
	*r* ^d^	*P* value	*r*	*P* value	*r*	*P* value		
Allergy	0.513	*<.001*	0.491	*<.001*	0.374	*<.001*	Yang et al [33]	Yes
Antidepressant	0.295	*.001*	0.291	*.001*	0.194	*.02*	Yang et al [33]	Insufficient
Alcohol abstinence	0.295	*.001*	0.251	*.003*	0.296	*.001*	Yang et al [33]	Yes
Relationship breakup	0.268	*.002*	0.191	*.02*	0.365	*<.001*	Yang et al [33]	No
Unemployed + lost job	0.253	*.003*	0.234	*.005*	0.206	*.012*	Yang et al [33]	No
Pain	0.239	*.004*	0.208	*.011*	0.230	*.006*	Yang et al [33]	No
Drunkenness	0.211	*.01*	0.142	.06	0.310	*<.001*	Yang et al [33]	No
Suicide	0.199	.02	0.133	.07	0.296	*.001*	McCarthy [8]; Yang et al [33]; Bruckner et al [36]; Sueki [32]	Yes; Yes; No; No
Insomnia	0.193	*.02*	0.160	*.04*	0.204	*.013*	Yang et al [33]	Yes
Suicide and depression	0.186	*.02*	0.116	.104	0.298	*<.001*	Bruckner et al [36]	Yes
Major depression	0.184	*.02*	0.118	.099	0.285	*.001*	Yang et al [33]	Yes
Headache	0.183	*.02*	0.163	*.04*	0.165	*.04*	Yang et al [33]	No
****								
A suicide	0.174	*.03*	0.145	*.06*	0.183	.02	Evans [37]	Yes
A suicide	0.171	*.03*	0.181	*.02*	0.079	.19	Yang et al [33]	No
Marriage	0.130	.08	0.083	.18	0.201	*.014*	Yang et al [33]	Yes
Anxiety disorder	0.126	.09	0.084	.18	0.185	*.02*	Yang et al [33]	Yes
Charcoal burning	0.120	.097	0.044	.32	0.271	*.001*	Yang et al [33]	Insufficient
Chronic illness	0.115	.11	0.097	.15	0.119	.098	Yang et al [33]	No
Lawsuit	0.115	.11	0.094	.15	0.124	.09	Yang et al [33]	Yes
Cancer	0.113	.11	0.093	.16	0.122	.09	Yang et al [33]	No
Hanging	0.110	.12	0.077	.20	0.154	*.046*	Yang et al [33]	Insufficient
Divorce	0.064	.24	0.028	.38	0.133	.07	Yang et al [33]	Yes
Suicide methods	0.042	.32	–0.005	.48	0.149	.053	Yang et al [36]; Evans [37]; Sueki [32]	No; No; No
Hypnotics	0.042	.33	0.015	.44	0.096	.15	Yang et al [33]	Insufficient
Depression	0.038	.34	0.001	.497	0.120	.096	Bruckner et al [36]; Sueki [32]	Yes; Yes
Commit suicide	0.032	.37	–0.015	.44	0.141	.06	Gunn and Lester [31]	Yes
Stress	0.010	.46	–0.043	.32	0.144	.06	Yang et al [33]	Yes
Hydrogen sulﬁde	0.009	.46	–0.022	.41	0.087	.17	Evans [37]	Yes
Teen suicide	–0.018	.42	–0.023	.40	0.002	.49	McCarthy [8]	Yes
Bipolar disorder	–0.029	.38	–0.051	.29	0.041	.33	Yang et al [33]	Yes
Schizophrenia	–0.042	.33	–0.098	.14	0.121	.09	Yang et al [33]	No
Social beneﬁts	–0.043	.32	–0.001	.495	–0.135	.07	Yang et al [33]	No
Domestic violence	–0.048	.30	–0.093	.16	0.089	.17	Yang et al [33]	Yes
Job	–0.048	.30	–0.068	.23	0.024	.40	Yang et al [33]	No
Asthma	–0.092	.16	–0.098	.14	–0.040	.33	Yang et al [33]	Yes
Abuse	–0.103	.13	–0.071	.22	–0.147	.06	Yang et al [33]	No
Taiwan economy	–0.105	.13	–0.114	.11	–0.043	.32	Yang et al [33]	Yes
Religious belief	–0.109	.12	–0.132	.08	–0.006	.47	Yang et al [33]	Yes
Alcohol	–0.113	.11	–0.132	.08	–0.019	.42	Yang et al [33]	No
Illicit drugs	–0.127	.08	–0.158	*.04*	0.003	.49	Yang et al [33]	No
Social welfare	–0.146	.06	–0.116	.10	–0.167	*.03*	Yang et al [33]	Yes
Stock market	–0.231	*.006*	–0.222	*.007*	–0.166	*.04*	Yang et al [33]	No

^a^GT: Google Trends.

^b^RSV: Relative Search Volume.

^c^INE: Spanish National Statistics Institute.

^d^Column from which the table is sorted.

**Table 4:** Predictive-explanatory model obtained by linear regression using as predictors search terms with a significance *P*<.05 and the variable suicide rates in Spain collected by the Spanish National Statistics Institute in the 2004-2013 period.

Predictors: search terms	*r*	*r* ^2^	*r*^2^ adjusted	Standard error of estimation	Beta (standard)	*t* test	*P* value
a: Allergy	0.513	0.264	0.257	28.2157	0.441	5.845	<.001
b: a + Relationship Breakup	0.552	0.304	0.292	27.5421	0.205	2.823	.006
c: b + Unemployment (paro)	0.583	0.340	0.322	26.9515	1.134	3.710	<.001
d: c + Headache	0.607	0.369	0.347	26.4618	–0.721	–3.279	.001
e: d + Unemployment (desempleo)	0.631	0.398	0.371	25.9586	–0.366	–2.294	.02
f: e + Antidepressant	0.647	0.418	0.387	25.6255	0.152	1.996	.048

### Results of Objective 2: Differences Between Men and Women

With regard to the second objective of our work, we found correlations between the terms of study and suicide rates between women and men (Table 5). To describe the strength of the correlation between our variables, we have used the interpretation by Evans [37]; as it can be seen in absolute terms, there is an important difference between the correlation of men and women.

The significant difference between male and female correlations can be explained by women’s use the internet for searching for health and lifestyle information. In contrast, men tend to focus on information about investment, purchase, and personal interests [40]. Moreover, this would be consistent with the idea that women have higher emotional intelligence and more communication skills than men [41].

**Table 5:** Number of positive (+) or negative (–) correlations found among the 41 Google search terms or phrases and suicide rates according to sex as reported by Spanish National Statistics Institute for the 2004-2013 period.

Correlation strength by gender	Evans’ scale	Total	Men	Women
Very weak or none	0.00-0.19	34	36	30
Weak	0.20-0.39	6+, 1–	4+, 1**­–**	12+
Moderate	0.40-0.59	1	1	0
Strong	0.60-0.79	0	0	0
Very strong	0.80-1.0	0	0	0

## Discussion

### Correlation Between Suicidal Rates and Internet Searches

It is not clear whether the information found on the internet contributes to the promotion of suicide and inspires suicidal thoughts or reduces the risk of suicidal behavior. The causal relationship between suicide and the use of the internet to search for topics related to self-harm or suicide is difficult to prove; however, the results of our study suggest a significant correlation for a number of the search terms that we have studied. This is consistent with previous studies that have been outlined throughout this paper that mostly state that there is indeed some association between certain searches and social phenomena in the economics, health, and other sectors.

Suicide rates in Spain for the 2004-2013 period were examined for their association with search volume on Google for 41 suicide-related searches already tested in scientific studies in other countries and languages. For the general population, suicide rates in Spain were positively associated with the search volume (*r*>0.2) for 7 terms and negatively associated with the search volume for 1 of the 41 terms. Our interpretation of the results is that they corroborate the hypothesis that certain searches on Google may serve as an indicator of a country’s suicide rate, perhaps even of its social well-being.

The negative correlations that we would call “protective” (for searches aimed at finding a solution to the problem) are interesting; the 41 searches in the original studies were supposed to be “risk related” in relation to suicide incidence, but one of them, *Stock Market* (*r*=–0.231; *P*=.006), correlated negatively along with some others that may have a "protective" significance (Social welfare, Religious belief, etc). As a preliminary explanation, we believe that this is due to the social and cultural translocation of the search terms, eg, in Spain unlike Taiwan—where the previous study for the search *Stock Market* was performed—only wealthy people are concerned about the topic.

In the case of *Drunkenness* (*r*=0.211; *P*=.01) versus the negative correlation for *Alcohol* (*r*=–0.113; *P*=.11), the latter could be interpreted as a protective search to find a solution to the problem, while the former may be used in a leisurely way (ie, without a problematic consciousness). This led us to an important point: Google Trends includes data from subjects with suicidal behavior searching in Google in addition to searches by other people concerned about the issue. We called these two perspectives as “first person” and “third person.” We then realized that Google Trends data includes “first person” searches from subjects with suicidal ideation and “third person” searches from their relatives, social surroundings, and institutions; therefore, it is crucial to try to segregate one from the other for future studies. Perhaps, this can be done with the help of linguistics differentiating the denotative aspects of words from their connotative aspects. 

### Comparison With Prior Work

The term that correlates more strongly with the overall rate of suicide is *Allergy* (*r*>0.5 and *P*<.001), which is consistent with other studies linking depression and allergy [42,43].

However, the overlap between the terms that correlate in our study and those that correlate in other studies is only about half. This could be due to cultural differences between the regions of the study subjects. It could also be due to semantic changes lost (or gained) in translation: Although the studies that we used to build our research were written in English, the original language of the study was Japanese or Mandarin Chinese in several cases, which resulted in two nested translations in this study.

Comparing our results for Spain with some of the results from a study by Yang et al [33] for Taiwan, there are differences that we can charge to cultural or sociological variances between the subjects of each study. Although in the Taiwanese study, the term *Divorce* correlates with suicide rates, in our study, it does not correlate with suicide rates. *Unemployed* does not correlate with suicide rates, according to Yang et al [33], but it does so in Spain, where unemployment is approximately 4 times higher than it is in Taiwan. We interpret these as indicators of issues that have a different significance and social context in these two regions. It should be noticed that while the search term *Divorce* (*r*=0.064; *P*=.24) does not correlate with suicide rates in Spain, *Relationship Breakup* (*r*=0.268; *P*=.002) does and this is the second strongest correlation among women. Further consideration on gender differences are made in the following section.

Another reason for these disparities might be deficiencies in the study methodology since using Google Trends as a diagnostic indicator of a society’s well-being is still fairly new.

Other interesting evidence our study demonstrates is that well-known risk factors (eg, depression) and explicit searches (eg, suicide) are not correlated with suicide rates; this could be interpreted as follows: the better the knowledge of the risk situation, the less likely it is that this risk of suicide will materialize.

### Differences Between Men and Women

Although there is no gender segregation in Google Trends RSV data, as Spanish suicide rates from the INE are segregated by gender, we were able to find differing gender-based correlations: We have found 5 terms that correlate for suicide rates among men and 12 terms in the case of women. Search terms that correlate with suicide rates of women are consistent with previous studies, showing that the incidence of depression is higher in women than in men [44].

In short, we have obtained more than twice the correlations between suicide rates for women compared with those obtained for men. We understand that this is due to the fact that patterns of internet usage among women are more oriented toward searches on health or lifestyle [40], which is also very much in line with the idea that women have more emotional intelligence than men [41]. This would explain why their suicide rate is much lower than that of men in Spain.

### Limitations

Owing to the limitations of evidence, we cannot actually predict increases of suicidal mortality using web search data. Rather, we undertook a preliminary investigation using the entire available dataset to establish a statistical association between search term usage and actual suicides in Spain. Further studies should compute time-lagged correlations between Google searches and suicides to help prevent suicide-related deaths.

### Social Applications

The practical implication of our results are as follows: It is desirable that competent authorities establish agreements with Google to facilitate suicide prevention by monitoring searches in Google for any of the terms that have been shown to correlate with suicide statistics and other terms that are proven to be significant in future studies.

We also hope that our research will help design and maintain websites that provide better education for suicide prevention, focusing on the treatment of depression and management of labor or emotional problems, as these fields show greater explanatory-predictive value in the incidence of suicide according to our regression model.

### Future Developments

An interesting avenue for future research on suicide-related searches is obtaining data from large social networks such as Facebook or Twitter, rather than just metasearch engines like Google.

In addition, the results of this study suggest the feasibility of using the Google search volume to predict other social risk behaviors such as traffic accidents, domestic violence, and bullying, and for the epidemiological monitoring of the evolution of emotional disorders in society. In general, we believe that tracking the search volumes of certain terms (eg, ones related to suicide) represents satisfaction in and well-being of a society. Hence, there may even be an application to the field of politics.

### Other Considerations

We want to point out some interesting facts that we have come across in our research and that we consider to be significant in correctly interpreting the world of big data and metasearch engines.

First, as mentioned earlier, it is interesting to note that despite the fact that more than 10,000 scientific papers used or mentioned the Google Trends service, according to Google Scholar, we did not find any mathematical formulation of how Google Trends operationalizes the values that it returns, which is the reason why we have developed it ourselves (Figure 1).

Another fact that seems significant is the case of GISI [20] or the Google Price Index (GPI), which are Google initiatives from 2010, that disappeared despite evidence of good results for forecasting phenomena. According to comments on web-based forums, Google chief economist, Dr Hal Varian, said that GPI was never intended be a project or public source of data; it was simply an internal Google project made visible by the press [45].

In any case, the opportunities and risks of using information from internet metasearches are yet to be determined; with this work, we hope to have contributed some clarity to this field of study.

## Abbreviations

GISI: Google Inflation Search Index
GPI: Google Price Index
INE: Spanish National Statistics Institute
RSV: Relative Search Volume
WHO: World Health Organization
